# Apigenin potentiates TRAIL therapy of non-small cell lung cancer *via* upregulating DR4/DR5 expression in a p53-dependent manner

**DOI:** 10.1038/srep35468

**Published:** 2016-10-18

**Authors:** Minghui Chen, Xueshi Wang, Daolong Zha, Fangfang Cai, Wenjing Zhang, Yan He, Qilai Huang, Hongqin Zhuang, Zi-Chun Hua

**Affiliations:** 1 The State Key Laboratory of Pharmaceutical Biotechnology, School of Life Sciences, Nanjing University, Nanjing 210023, China; 2 State Key Laboratory of Quality Research in Chinese Medicines, Macau University of Science and Technology, Avenida Wai Long, Taipa, Macau; 3 Changzhou High-Tech Research Institute of Nanjing University and Target pharma Laboratory, Changzhou 213164, Jiangsu, China; 4 College of Pharmacy, Nanjing University of Chinese Medicine, Nanjing 210046, China

## Abstract

Apigenin (APG) is an edible plant-derived flavonoid that shows modest antitumor activities *in vitro* and *in vivo*. APG treatment results in cell growth arrest and apoptosis in various types of tumors by modulating several signaling pathways. In the present study, we evaluated interactions between APG and TRAIL in non-small cell lung cancer (NSCLC) cells. We observed a synergistic effect between APG and TRAIL on apoptosis of NSCLC cells. A549 cells and H1299 cells were resistant to TRAIL treatment alone. The presence of APG sensitized NSCLC cells to TRAIL-induced apoptosis by upregulating the levels of death receptor 4 (DR4) and death receptor 5 (DR5) in a p53-dependent manner. Consistently, the pro-apoptotic proteins Bad and Bax were upregulated, while the anti-apoptotic proteins Bcl-xl and Bcl-2 were downregulated. Meanwhile, APG suppressed NF-κB, AKT and ERK activation. Treatment with specific small-molecule inhibitors of these pathways enhanced TRAIL-induced cell death, mirroring the effect of APG. Furthermore, using a mouse xenograft model, we demonstrated that the combined treatment completely suppressed tumor growth as compared with APG or TRAIL treatment alone. Our results demonstrate a novel strategy to enhance TRAIL-induced antitumor activity in NSCLC cells by APG *via* inhibition of the NF-κB, AKT and ERK prosurvival regulators.

Based on the World Health Organization (WHO) mortality database, lung cancer is the most common neoplasm in human in both developed and developing countries^1^,^2^, and non-small cell lung cancer (NSCLC) accounts for approximately 75–85% of all cases^3^,^4^. Despite recent improvements in diagnosis and first-line treatment, prognosis remains very poor, with an overall 5-year survival probability of about only 15%. Since current treatment modalities are still far from optimal results, novel therapies are needed to reduce the effects of the increasing incidence in pulmonary neoplasm.

Tumor necrosis factor-related apoptosis-inducing ligand (TRAIL; also known as apo2 ligand) is a member of the TNF family that triggers rapid apoptosis *in vitro* and *in vivo* in various tumor cells while leaving most normal cells unscathed^5^. TRAIL induces apoptosis *via* interacting with death receptor 4 (DR4) and death receptor 5 (DR5), which leads to the formation of the death-inducing signaling complex (DISC) with subsequent binding of caspase-8. Recruitment of caspase-8 to the DISC activates its proteolytic properties, which initiates a cascade of protease such as caspase-3, promoting the cleavage of death substrates and finally resulting in apoptosis. Because TRAIL can induce apoptosis in cancer cells but has little effect on normal cells, it is considered as a promising anticancer agent^6^. However, although many tumors are sensitive to TRAIL-mediated apoptosis, the majority, including lung cancer, remains resistant^7^,^8^. This resistance is conferred by a number of molecular changes, such as the differential expression of death receptors; the elevated expression of anti-apoptotic molecules, including FLICE-like inhibitory protein (FLIP), X-linked inhibitors of apoptosis proteins (XIAPs), anti-apoptotic Bcl-2-family proteins; and the activation of AKT and NF-κB in resistant cells^9^. In fact, combination therapies using recombinant TRAIL with other anti-cancer agents have shown improved efficacy for cancer treatment *in vitro* and *in vivo* through modulation of TRAIL-resistant mechanisms^9^.

Flavonoids plentifully contained in fruits and vegetables are a class of plant secondary metabolites with a ubiquitous phenolic structure. Current trends in cancer research show that flavonoids are highly favorite plant-derived compounds for using alone or in combination with another therapeutic agent for controlling the growth of various malignant cells both *in vitro* and *in vivo*. Apigenin (4′,5,7-trihydroxyflavone; APG; Fig. 1a), a type of flavonoid found mainly in orange, chamomile tea, onion, and wheat sprouts, has a potential for biological activities, including antioxidant and anti-inflammatory. APG has recently received much attention, due to its strong anticancer effect in various cancer cells, including breast cancer, colon cancer, lung cancer, neuroblastoma, liver cancer, prostate cancer, pancreas cancer, and oral cancer cells^10^,^11^. There are several proposed mechanisms for the anticancer effects of APG. Previous reports revealed that APG could induce cell cycle arrest and enhance apoptosis in cancer cells and xenograft models^12^,^13^. Moreover, APG has been shown to downregulate NF-κB activity through the suppression of phosphorylation of p65^14^ and AKT signaling may be responsible for these processes^15^. In addition, it was demonstrated that APG exerted potent cancer chemopreventive activities through regulating the intracellular accumulation of reactive oxygen species (ROS) and antioxidant enzyme expression in lung cancer cells^16^. However, due to low efficacy of this compound, the clinical use of APG is relatively restricted.

Recently, combined therapy using APG with other chemotherapeutic drugs has been shown to enhance anticancer effects in various tumors^15^,^17^. The combined inhibitory effects of APG and other chemotherapeutic agents on tumor cell growth were reported to be superior to the effects of these agents used alone. Chan *et al*.^17^ demonstrated that APG could induce cell apoptosis *via* tumor necrosis factor receptor (TNF-R)-, and TNF-related apoptosis-inducing ligand receptor (TRAIL-R)-mediated caspase-dependent cell death pathways in tumor cells. These findings led us to hypothesize that the combined treatment with APG might enhance the cytotoxic effect of TRAIL on NSCLCs. Thus, this present study aims to explore the anti-tumor ability of APG with TRAIL, using both NSCLC cells and a xenograft mouse model, besides the investigation of potential mechanisms of action.

## Results

### Combined effect of APG and TRAIL on growth of tumor cells

**Figure 1:**
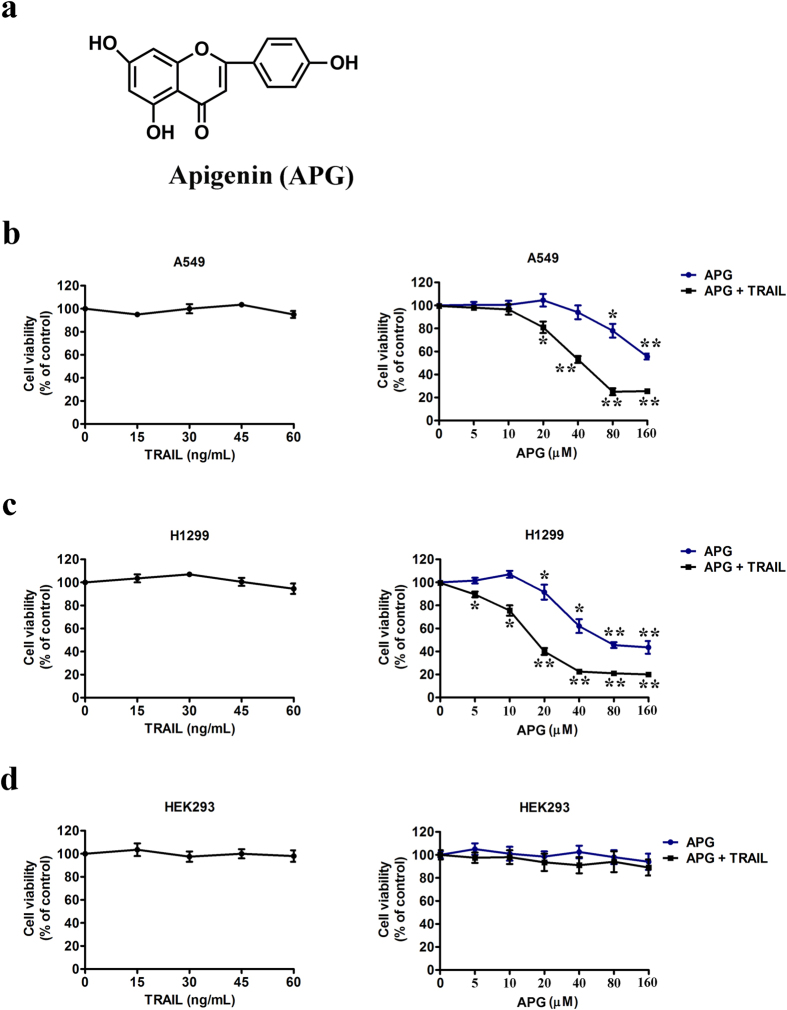
APG enhances TRAIL-induced cell proliferation inhibition of NSCLC cells. (**a**) Chemical structure of APG. (**b–d**) Left, A549 cells (**b**), H1299 cells (**c**) and HEK293 cells (**d**) were treated with TRAIL at different concentrations (0, 15, 30, 45, 60 ng/mL) for 24 h, and the cell viability was assessed by MTT assay. Right, A549 cells (**b**), H1299 cells (**c**) and HEK293 cells were treated with TRAIL at the concentration of 25 ng/mL for 24 h in the presence of indicated concentrations of APG. The cell viability was assessed by MTT assay. Data are represented as mean ± SD. *p < 0.05, **p < 0.01.

Before testing the combined effect of APG and TRAIL therapy, we first evaluated the cytotoxicity of TRAIL monotherapy in two NSCLC cell lines, A549 and H1299, and a normal cell line HEK293, by means of MTT assay. Our data showed that, at the concentration of 60 ng/mL or lower, TRAIL showed no significant antitumor effect on A549 and H1299 cells, indicating that both NSCLC cell lines had low sensitivity or were resistant to TRAIL monotherapy (Fig. 1b,c). In order to assess the combined effect of APG and TRAIL on tumor cell proliferation, MTT assay was also performed. Two lung cancer cell lines were treated with the indicated concentrations of APG alone and its combination with 25 ng/mL of TRAIL (APG + TRAIL) for 24 h. The results showed that APG alone promoted decreased cell viability in a dose-dependent manner, while APG + TRAIL showed the strongest anti-proliferation ability, which surpassed the sum effect of APG (Fig. 1b,c). At the concentration of 10 μM or lower, no significant difference of the inhibition rate between groups treated with APG + TRAIL and APG was found. While at the drug concentration of 20 μM, APG + TRAIL showed huge anti-proliferation ability on A549 cells, with the inhibition rate larger than that of APG (*p* < 0.05) (Fig. 1b). Compared to A549 cells, H1299 cells were more sensitive to APG + TRAIL treatment, once the proliferation inhibition rate was 25.7 ± 2.9 (%) since 10 μM of APG (Fig. 1c). In contrast, no synergistic cytotoxicity was observed in HEK293 cells (Fig. 1d). These results suggest that APG at a subtoxic concentration has an enhanced effect on TRAIL-inhibited proliferation of tumor cells without increasing cytotoxicity to normal cells. In this study, we used the highest concentration at which APG alone did not induce significant proliferation inhibition. Therefore, drug concentrations of APG (20 μM) and TRAIL (25 ng/mL) were chosen for combined therapy in the following experiments.

### APG sensitizes NSCLC cell lines to TRAIL-induced apoptosis

**Figure 2:**
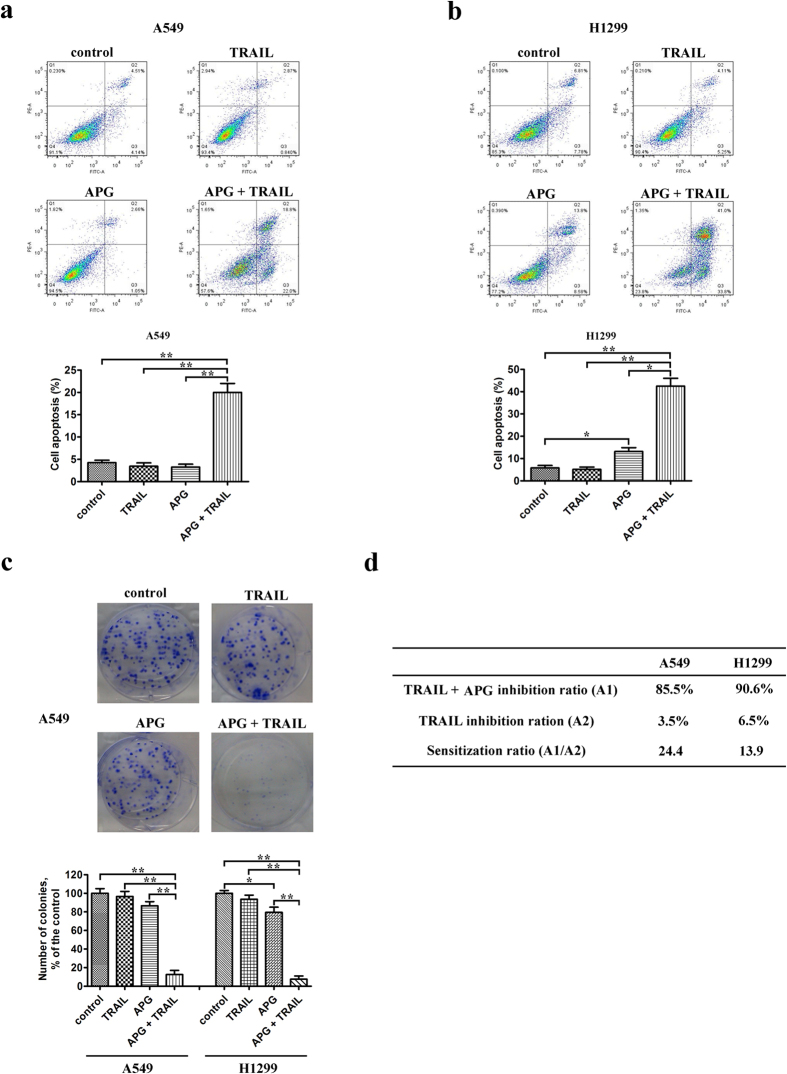
APG enhances TRAIL-induced cell apoptosis and the combination of two drugs suppresses the clonogenic growth of NSCLC cells. (**a,b**) A549 (**a**) and H1299 (**b**) cells were exposed to APG (20 μM) and/or TRAIL (25 ng/mL). Eighteen hours later, all cells were harvested for flow cytometry analysis. Annexin V/PI-stained cells were analyzed and the percentage of apoptotic cells was determined. The experiments were carried out independently in triplicate; representative data are shown. Data are represented as mean ± SD. *p < 0.05, **p < 0.01. (**c**) Colony formation ability of NSCLC cells treated with APG (20 μM) and/or TRAIL (25 ng/mL). The experiments were repeated three times (n = 3); representative data are shown. Data are represented as mean ± SD. *p < 0.05, **p < 0.01. Representative dishes of A549 cells evaluated by colony-forming assay are also included. (**d**) Sensitization ratio for NSCLC cells to TRAIL-induced apoptosis by APG treatment.

We next investigated whether APG treatment could sensitize NSCLC cells to TRAIL-induced apoptosis. The flow cytometric studies showed that the combination of 20 μM APG and 25 ng/mL TRAIL caused an apparent increase in the apoptotic rate of treated A549 and H1299 cells as compared to TRAIL or APG monotherapy (Fig. 2a,b). However, combined treatment with APG and TRAIL showed no synergistic effect on the apoptotic rate of HEK293 cells (Supplementary Fig. S1). DAPI staining further showed that APG treatment resulted in an increased apoptotic rate in NSCLC cells co-treated with TRAIL (Supplementary Fig. S2). Nuclear condensation is a typical morphology change of apoptotic cells, which can be detected by DAPI staining even in early stage of cell death^18^. As shown in Supplementary Fig. S2a, most of the H1299 cells in the control group emitted uniform blue color, where 9.1 ± 1.5 (%) of which exhibited condensed nuclei (naturally apoptotic cells). In APG-treated cells, 21.4 ± 3.7 (%) presented condensed stained nuclei. In TRAIL treatment group, 18.2 ± 2.4 (%) of the observed cells showed stained depressed nuclei. However, nuclear condensation and chromatin margination were typical and commonly observed in the group of APG + TRAIL, where 58.5 ± 4.1 (%) of the treated cells showed apoptotic morphology. These data indicated that the capability of APG + TRAIL to induce H1299 cell apoptosis was significantly stronger than the sum effect of APG or TRAIL separately used in resembling experiments. Similar synergistic effects of APG and TRAIL were also found in A549 cells (Supplementary Fig. S2b).

### Combined effect of APG and TRAIL suppresses the clonogenic growth of NSCLC cells

We were interested in whether the treatment of APG affected the clonogenic growth of NSCLC cells upon simultaneous exposure to TRAIL. Our colony-forming assays showed that 20 μM APG or 25 ng/mL TRAIL alone caused minimal inhibition of the clonogenic growth of A549 and H1299 cells (Fig. 2c). In contrast, combined treatment with APG and TRAIL markedly suppressed the clonogenic growth of A549 and H1299 cells with inhibition rates of 85.5% and 90.6%, respectively (Fig. 2d). The sensitization ratios for A549 and H1299 cells to TRAIL-induced apoptosis by APG treatment were 24.4 and 13.9, respectively (Fig. 2d).

### APG sensitizes A549 cells to TRAIL-induced apoptosis through the caspase-dependent mitochondrial pathway

**Figure 3:**
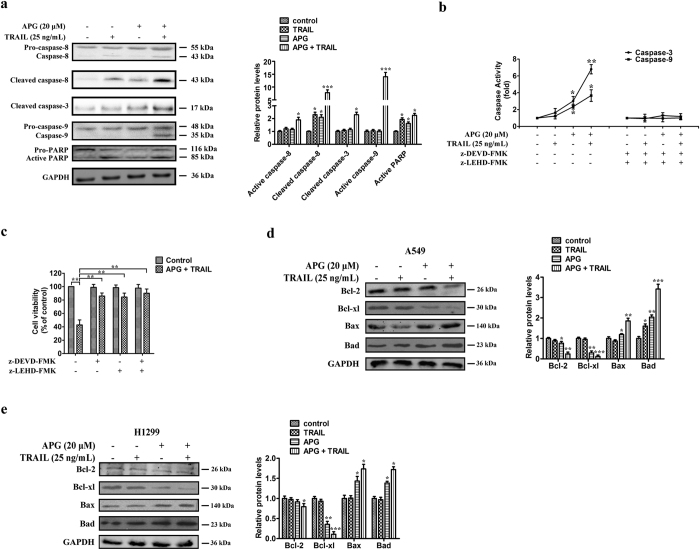
Sensitization of TRAIL-induced apoptosis by APG treatment is mediated through the caspase-dependent mitochondrial pathway in NSCLC cells. (**a**) Caspase-8, caspase-9, caspase-3, and PARP expression levels in A549 cells under different treatment conditions. All gels run under the same experimental conditions and the representative images of three different experiments were cropped and shown. Densitometric quantification of the immunoblot data is also shown and data are represented as mean ± SD. *p < 0.05, ***p < 0.001. (**b**) Activity of caspase-3 and caspase-9 in A549 cells treated with APG and TRAIL alone or in combination for 24 h. Data are presented as fold increases as determined by quantitative analysis. *p < 0.05, **p < 0.01. (**c**) Viability of A549 cells after treatment with caspase inhibitors. Cells were treated with inhibitors for 2 h before the 24 h treatments, after which cell viability was determined by MTT assay. Data are representative of three independent experiments and are represented as mean ± SD. **p < 0.01. (**d,e**) Expression levels of the Bcl-2 family proteins, Bcl-2, Bcl-xl, Bax, and Bad, in A549 (**d**) and H1299 (**e**) cells under different treatment conditions. All gels run under the same experimental conditions and the representative images of three different experiments were cropped and shown. Densitometric quantification of the immunoblot data is also shown and data are represented as mean ± SD. *p < 0.05, **p < 0.01, ***p < 0.001.

TRAIL-induced activation of caspase-8 leads to the activation of downstream caspase-9 and caspase-3. To explore the mechanisms of APG and TRAIL-induced apoptosis in A549 cells, we determined the activation of caspases in TRAIL-resistant A549 cells treated with 25 ng/mL TRAIL in the absence or the presence of APG. Western blotting assays showed that APG and TRAIL by themselves caused minimal proteolytic processing of procaspase-8, procaspase-9, and procaspase-3. In contrast, APG in combination with TRAIL caused an apparent more intensive proteolytic cleavage of procaspase-8, procaspase-9, and procaspase-3 (Fig. 3a). Moreover, the combined treatment with APG and TRAIL resulted in the cleavage of PARP, whereas APG or TRAIL alone failed to induce PARP cleavage (Fig. 3a). Caspase activity, shown in Fig. 3b, indicated that caspase-3 and caspase-9 activities were respectively 1.6- and 1.2-fold elevated in relation to controls in TRAIL-treated cells and respectively 6.8- and 3.7-fold over that in combined treatment. Co-treatment with the caspase inhibitors z-DEVD-FMK and z-LEHD-FMK abolished caspase activation induced by APG and TRAIL and rescued A549 cells from treatment-induced cell death (Fig. 3b). Cell viability was also increased by caspase inhibitors after combined treatment (Fig. 3c). These findings indicate that activation of a caspase-involved apoptotic pathway is one of the major mechanisms *via* which APG exerts its synergistic effect on TRAIL-treated A549 cells.

We further examined the effect of APG and TRAIL combined therapy on the balance between the apoptotic (Bax or Bad) and anti-apoptotic (Bcl-2 or Bcl-xl) members of the Bcl-2 family in A549 and H1299 cells. We found that the combined treatment of NSCLC cells with APG and TRAIL noticeably increased the levels of Bax and Bad with a prominent reduction of Bcl-2 and Bcl-xl levels (Fig. 3d,e). The mRNA levels of Bcl-2 and Bcl-xl were also decreased significantly in NSCLC cells treated with the combination of APG and TRAIL (Supplementary Fig. S3). These results suggest that APG sensitizes A549 and H1299 cells to TRAIL-induced apoptosis by the caspase-dependent mitochondrial pathways.

### The combination of APG and TRAIL inhibits the NF-κB p65 nuclear translocation, the IκB-α degradation, the PI3K/AKT cascade and activates the JNK-c-JUN pathway

**Figure 4:**
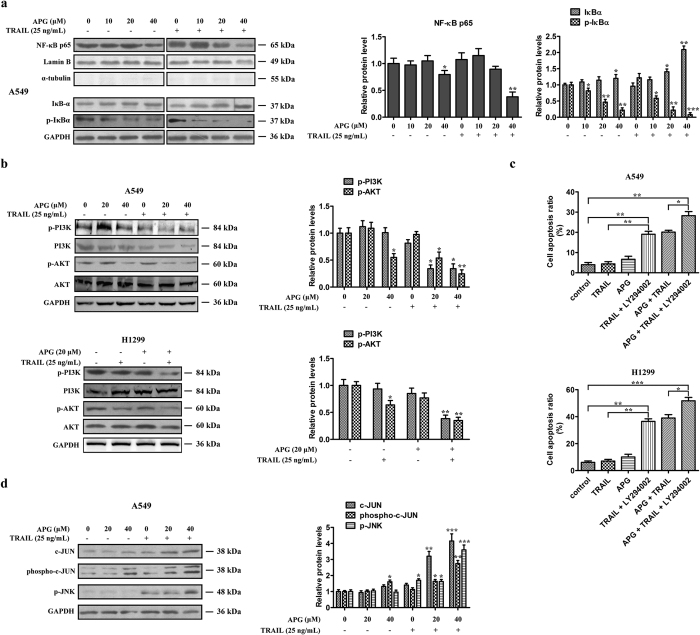
Effects of APG and TRAIL on NF-κB, PI3K/AKT and JNK signaling pathway. (**a**) A549 cells were treated with APG at different concentrations (0, 10, 20, 40 μM) in the absence or the presence of TRAIL (25 ng/mL) for 24 h. Nuclear proteins were extracted and subjected to Western blotting for p65 detection. Lamin B was used as loading control. Additionally, the whole cell extracts with the same treatment were prepared and analyzed for IκB-α and p-IκB-α expression. (**b**) A549 and H1299 cells were treated with APG at different concentrations (0, 20, 40 μM) in the absence or the presence of TRAIL (25 ng/mL) for 24 h. AKT, p-AKT, PI3K, and p-PI3K proteins in whole cell lysates were determined with specific antibodies. GAPDH was used as loading control. (**c**) A549 and H1299 cells were treated with APG (20 μM), TRAIL (25 ng/mL), LY294002 (5 μM), or their combination for 24 h before determination of cell death by flow cytometry analysis. The experiments were carried out independently in triplicate; representative data are shown. Data are represented as mean ± SD. *p < 0.05, **p < 0.01, ***P < 0.001. (**d**) A549 cells were treated with APG at different concentrations (0, 20, 40 μM) in the absence or the presence of TRAIL (25 ng/mL) for 24 h. c-JUN, p-c-JUN and p-JNK protein in whole cell lysates were determined with specific antibodies. GAPDH was used as loading control. For (**a**,**b**,**d**), all gels run under the same experimental conditions and the representative images of three different experiments were cropped and shown. Densitometric quantification of the immunoblot data is also shown and data are represented as mean ± SD. *p < 0.05, **p < 0.01, ***p < 0.001.

NF-κB is generally considered to be a survival factor that activates expression of various anti-apoptotic genes, e.g. Bcl-2, Bcl-xl, Mcl-1 and c-FLIP that block apoptosis^19^. Inhibition of NF-κB will lead to downregulation of the NF-κB-regulated anti-apoptotic proteins thereby promoting apoptotic cell death. Thus, we investigated whether APG at different dosages was able to modify the rate of NF-κB inhibition. We found that low dosage of APG or TRAIL alone had no obvious effect on the expression of NF-κB/p65. However, in the analysis of nuclear extracts by western blotting, treatment of APG at the dosage of 40 μM in A549 cells inhibited the translocation of p65 from the cytoplasm to the nucleus, which was further accentuated upon exposure to TRAIL (Fig. 4a), probably leading to the inhibition of the transactivation of NF-κB target genes, such as Bcl-2 and Bcl-xl (Supplementary Fig. S3). Additionally, 40 μM APG treatment resulted in increased IκBα levels in A549 cells, which was further enhanced by concurrent exposure to TRAIL (Fig. 4a). APG also inhibited the phosphorylation of IκBα in cytosol extracts, suggesting that it abrogates the dissociation of IκBα from NF-κB heterodimer (p65 and p50) and blocks the proteasomal degradation of IκBα (Fig. 4a). In contrast, the combined treatment with APG and TRAIL had little effect on the nuclear translocation of p65 in HEK293 cells (Supplementary Fig. S4). c-FLIP, one of the targeted genes of NF-κB, is known to interfere with caspase activation downstream of death receptors. To evaluate the combined effect of APG and TRAIL on c-FLIP expression, we treated A549 cells with TRAIL in the absence or the presence of APG. Our quantitative RT-PCR (Q-PCR) assay showed that combined treatment decreased c-FLIP expression significantly, while TRAIL alone had no effect on c-FLIP expression (Supplementary Fig. S5).

AKT has been reported to suppress apoptosis by stimulating the transactivation potential of the RelA/p65 subunit of NF-κB^20^. As reported previously, the PI3K/AKT pathway was inhibited by APG in leukemia cells^21^. To figure out whether the AKT pathway was affected by APG and played a role in APG and TRAIL-induced cell death in NSCLC cells, A549 cells were treated with increasing doses of APG for 24 hours. As shown in Fig. 4b, the PI3K/AKT pathway was inhibited in proportion to the growing dosage of APG in A549 cells, which was further enhanced by concurrent exposure to TRAIL. H1299 cells were also exposed to APG and TRAIL alone or in combination for 24 hours. Figure 4b revealed that the phosphorylation levels of AKT and PI3K were markedly decreased after co-treatment with APG and TRAIL, but not with either drug alone. Flow cytometric studies further showed that the apoptosis of A549 and H1299 cells induced by the APG + TRAIL treatment was enhanced by the addition of LY294002 (5 μM), a PI3K/AKT inhibitor (Fig. 4c). LY294002 also dramatically enhanced TRAIL-induced cell death in both cell lines (Fig. 4c).

JNK is known to promote apoptosis by many cellular stresses, including oxidative stresses, and DNA-damaging agents^22^ and plays important roles in cell proliferation and apoptosis^23^. We hypothesized that JNK might be activated by cellular stress induced by APG and TRAIL combined treatment. As expected, the level of phospho-JNK increased in A549 cells co-treated with APG and TRAIL (Fig. 4d). Furthermore, the combination of APG and TRAIL induced an apparent increase in the level of phospho-c-JUN in A549 cells. In contrast, low dosage of APG (20 μM) failed to activate the JNK-c-JUN pathway, whereas TRAIL alone raised the phosphorylation of JNK, but not that of c-JUN, in relation to control (Fig. 4d). Taken together, these findings suggest that APG and TRAIL cooperatively induce apoptosis through the suppression of NF-κB transcriptional activity, inhibition of PI3K/AKT cascade and activation of JNK-c-JUN pathway.

### APG sensitizes NSCLC cells to TRAIL-induced apoptosis by upregulating DR4 and DR5 levels in a p53-dependent manner

**Figure 5:**
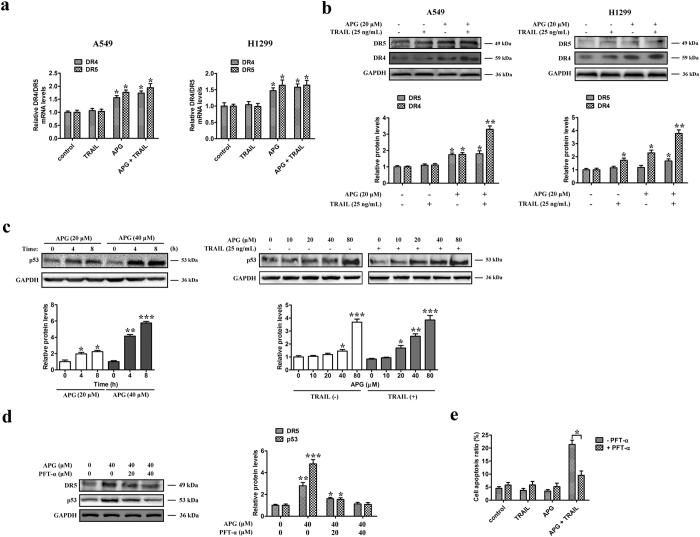
APG treatment sensitizes NSCLC cells to TRAIL-induced apoptosis by upregulating DR4 and DR5 levels in a p53-dependent manner. A549 cells and H1299 cells were treated with APG (20 μM) in the absence or the presence of TRAIL (25 ng/mL) for 24 h. (**a**) Q-PCR analysis was performed to detect the level of the mRNA transcripts of DR4 and DR5. The results shown are representative of three independent experiments. The histogram shows the mean ± SD. *p < 0.05: significantly different from the control. (**b**) Western blotting was performed to detect the levels of DR4 and DR5, respectively. (**c**) Left, A549 cells were treated with APG (20 μM or 40 μM) for the indicated time (4 h or 8 h). The protein levels of p53 in whole cell lysates were determined with specific antibody. Right, A549 cells were treated with APG at different concentrations (0, 10, 20, 40, 80 μM) in the absence or the presence of TRAIL (25 ng/mL) for 24 h. The protein levels of p53 in whole cell lysates were determined with specific antibody. (**d**) A549 cells were treated with APG or PFT-α at the indicated concentrations for 24 h. DR5 and p53 protein levels in whole cell lysates were determined with specific antibodies. For (**b**–**d**), all gels run under the same experimental conditions and the representative images of three different experiments were cropped and shown. Densitometric quantification of the immunoblot data is also shown and data are represented as mean ± SD. *p < 0.05, **p < 0.01, ***p < 0.001. (**e**) A549 cells were treated with APG (20 μM), TRAIL (25 ng/mL), or the combination of two drugs in the absence or presence of PFT-α (20 μM) for 18 h before determination of cell death by flow cytometry analysis. Data are representative of three independent experiments and are represented as mean ± SD. *p < 0.05.

Two TRAIL receptors, DR4 and DR5, which contain functional death domains, could trigger apoptotic signals upon TRAIL binding^24^. Our Q-PCR assays showed that APG by itself or in combination with TRAIL significantly increased the mRNA transcript levels of both DR4 and DR5 in A549 and H1299 cells (Fig. 5a). Immunoblotting assays further revealed that APG alone or in combination with TRAIL significantly upregulated the expression of DR5 in both NSCLC cell lineages (Fig. 5b). Furthermore, APG caused an approximately twofold increase in the levels of DR4 and, in combination with TRAIL, an approximately threefold increase in DR4 levels in A549 cells (Fig. 5b, left). Similarly, the combination of APG and TRAIL significantly elevated the expression levels of DR4 and DR5 in H1299 cells (Fig. 5b, right). However, all the treatments had no obvious effects on the expression of DR4 and DR5 in HEK293 cells (Supplementary Fig. S6). These results suggest that the upregulation of DR4 and DR5 levels may contribute to the increased sensitivity of A549 and H1299 cells to APG and TRAIL-induced apoptosis.

The p53 protein is known to play an important role in regulating the expression of DR4 and DR5 at the transcriptional level^25^. Our immunoblotting studies indicated that, consistent with the upregulation of DR4 and DR5 expression, APG by itself or in combination with TRAIL resulted in increased level of p53 protein (Fig. 5c). Moreover, following APG treatment, p53 protein levels were elevated in both a concentration- and time-dependent manner (Fig. 5c). To further determine the role of p53 in the synergistic apoptotic effect of APG and TRAIL, we evaluated the effect of the p53-specific inhibitor, PFT-α. As shown in Fig. 5d, immunoblotting analysis showed that the impact of APG on p53 and DR5 expression was reversed by PFT-α pre-treatment. Furthermore, pre-treatment of cells with PFT-α markedly reduced APG and TRAIL-induced apoptosis as compared with the untreated group (Fig. 5e). These results suggest that the apoptotic effect of the combination of APG and TRAIL is due to the activation of the p53 signaling pathway and the subsequent upregulation of DR4 and DR5 expression.

### APG inhibition of ERK contributes to synergistic interaction with TRAIL

**Figure 6:**
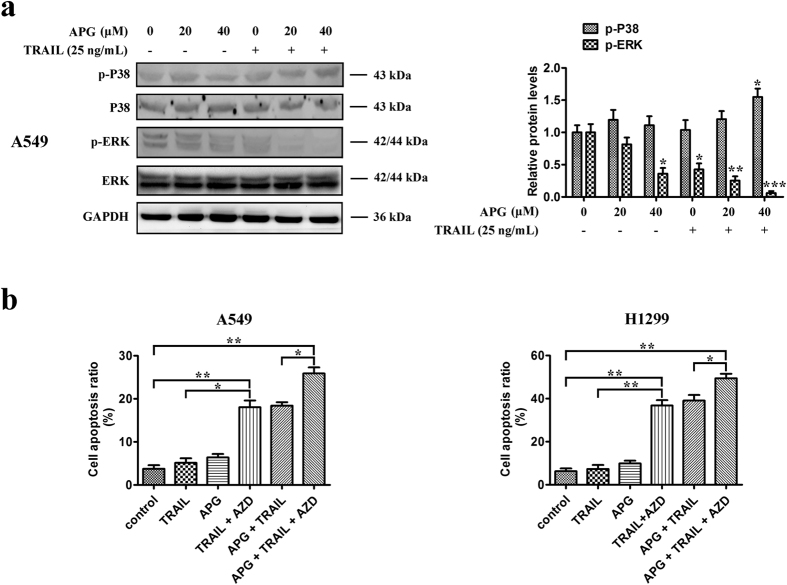
Effects of APG and TRAIL on ERK pathway. (**a**) A549 cells were treated with APG at different concentrations (0, 20, 40 μM) in the absence or the presence of TRAIL (25 ng/mL) for 24 h. Western blotting was performed to detect the levels of P38, p-P38, ERK, p-ERK, respectively. All gels run under the same experimental conditions and the representative images of three different experiments were cropped and shown. Densitometric quantification of the immunoblot data is also shown and data are represented as mean ± SD. *p < 0.05, **p < 0.01, ***p < 0.001. (**b**) A549 and H1299 cells were treated with APG (20 μM), TRAIL (25 ng/mL), AZD6244 (AZD; 2 μM), or their combination for 24 h before determination of cell death by flow cytometry analysis. Data are representative of three independent experiments and are represented as mean ± SD. *p < 0.05, **p < 0.01.

To clarify the effect of APG and TRAIL on MAPKs, cells were treated with APG at 20 or 40 μM for 48 h in the absence or presence of TRAIL, and ERK and p38 protein levels were measured. As shown in Fig. 6a, APG alone decreased ERK phosphorylation levels in a dose-dependent manner in A549 cells. This effect of APG remained significant when A549 cells were co-treated with TRAIL. In addition, APG in combination with TRAIL enhanced phospho-p38 protein levels. Consistent with inhibition of ERK contributing to the enhancement of TRAIL-induced cytotoxicity by APG, blocking ERK activation by AZD6244, a MEK inhibitor^26^, augmented the proapoptotic activity of TRAIL and sensitized A549 and H1299 cells to TRAIL-induced cell death (Fig. 6b). Thus, activation of the stress-response MAPK p38 and suppression of the survival MAPK ERK in A549 and H1299 cells may also account for the apoptotic effect of the combination of APG and TRAIL.

### Combined treatment of APG and TRAIL results in downregulation of HSP70

Previous studies have shown that HSP70 suppression or downregulation might be promising to overcome TRAIL resistance in cancer^27^. Thus, to clarify the effect of APG and TRAIL on HSP70 expression, cells were treated with APG at 20 or 40 μM for 24 h in the absence or presence of TRAIL, and HSP70, HSP27 and HSP90 protein levels were measured. As shown in Supplementary Fig. S7a, APG alone had no effect on the expression of HSP70; however, the combination of two drugs significantly decreased the protein level of HSP70. Furthermore, the combined treatment did not affect the expression of HSP27 and HSP90. Consistently, APG and TRAIL in combination resulted in the cleavage of PARP and decrease of Bcl-2 expression. In addition, flow cytometric studies further showed that APG in combination with TRAIL induced significant apoptosis of NSCLC cells, which was enhanced by the addition of 5 μM PFT-μ, a HSP70 inhibitor (Supplementary Fig. S7b). PFT-μ also augmented the proapoptotic activity of TRAIL and sensitized NSCLC cells to TRAIL-induced cell death. These results suggest that inhibition of HSP70 may be an effective approach for anticancer efficacy improvement of APG and TRAIL over lung cancer.

### APG increases TRAIL sensitivity of subcutaneous lung cancer xenografts, thereby significantly inhibiting tumor growth *in vivo*

**Figure 7:**
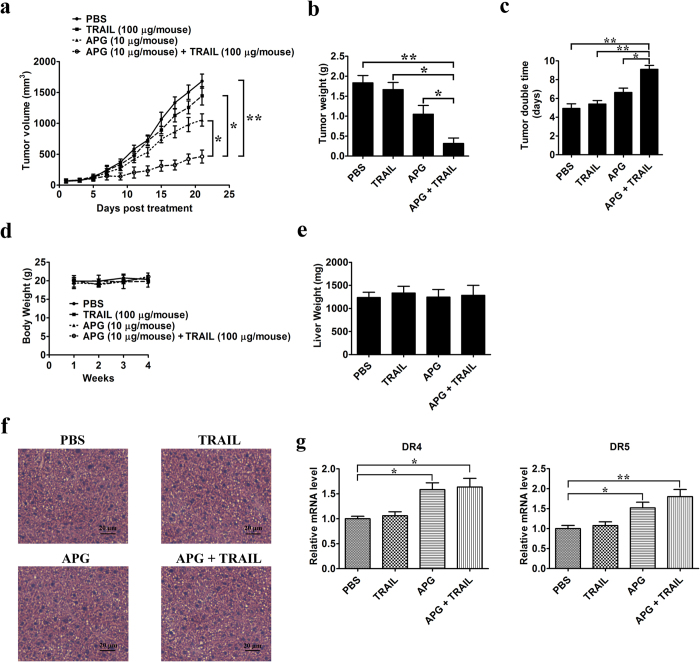
APG and TRAIL combined therapy inhibits *in vivo* tumor xenograft growth in a subcutaneous tumor model. A549 cells were injected subcutaneously into the dorsal flanks of athymic nude mice. When tumors reached a size of approximately 50 mm^3^, mice were i.p. with APG and TRAIL or the combination of two drugs every two day for a total of 21 days. (**a**) The tumor growth inhibitory effects of different treatments were compared. (**b**) At the end of the study, the excised tumors from each group were weighed. (**c**) Tumor double time of each group. (**d**) The weight of nude mice from each group did not change significantly during the experiment. (**e**) Liver weight of mice at the end of the experiment show in (**a**). (**f** ) Representative photomicrographs of liver sections stained with H&E of mice treated with PBS, TRAIL, APG, or APG + TRAIL. (**g**) Expression of TRAIL receptors DR4 and DR5 in tumors after APG and TRAIL treatment. Tumors were isolated on day 40 after tumor challenge and subjected to Q-PCR assay using primers specific for human DR4, DR5, and GAPDH (internal control). All data are shown as mean ± SD. *p < 0.05, **p < 0.01.

The antitumor effect of APG in combination with TRAIL was analyzed in a xenograft tumor model by transplanting A549 cancer cells into athymic nude mice. On the 8^th^ day post-implantation, mice were randomly divided into 4 groups before the tumor was palpated, with at least 8 tumor-bearing mice in each group. Tumor volume was significantly reduced after intraperitoneally injection of APG and TRAIL for 21 days as compared to APG or TRAIL monotherapy (Fig. 7a). APG monotherapy also inhibited the growth of xenograft tumors to some extent, but the effects were not as significant as those seen in the combined treatment group. At the end of the study, we removed the tumors and measured their weight for each group. Combined treatment with APG and TRAIL clearly reduced tumor weight compared with the control group, APG or TRAIL single treatment (Fig. 7b). Tumor doubling time was prolonged from 4.93 days in mice receiving PBS, 5.45 days in mice receiving TRAIL, 6.63 days in mice receiving APG to 9.25 days in mice receiving APG + TRAIL (CI = 1.95; Fig. 7c), indicating a supra-additive or synergistic effect of APG and TRAIL. In addition, the treatment of mice with APG alone, TRAIL alone, or APG and TRAIL combination induced no apparent toxicity and we noted no change in their behavior, body mass (Fig. 7d), or liver mass (Fig. 7e), suggesting an absence of hepatomegaly. We also evaluated the toxicity in the liver of mice with different treatments. H&E staining of the liver of mice treated with TRAIL, APG or APG + TRAIL showed normal histology when compared with control mice (Fig. 7f), indicating an absence of hepatic toxicity induced APG or TRAIL. Moreover, Q-PCR assays revealed that the xenografts of mice receiving APG, or the combination of APG and TRAIL, showed upregulated levels of DR4 and DR5 (Fig. 7g). These findings suggest that the TRAIL-sensitizing effect of APG on lung cancer cells observed *in vitro* also occurs *in vivo via* the same mechanism involving the regulation of TRAIL receptors.

**Figure 8:**
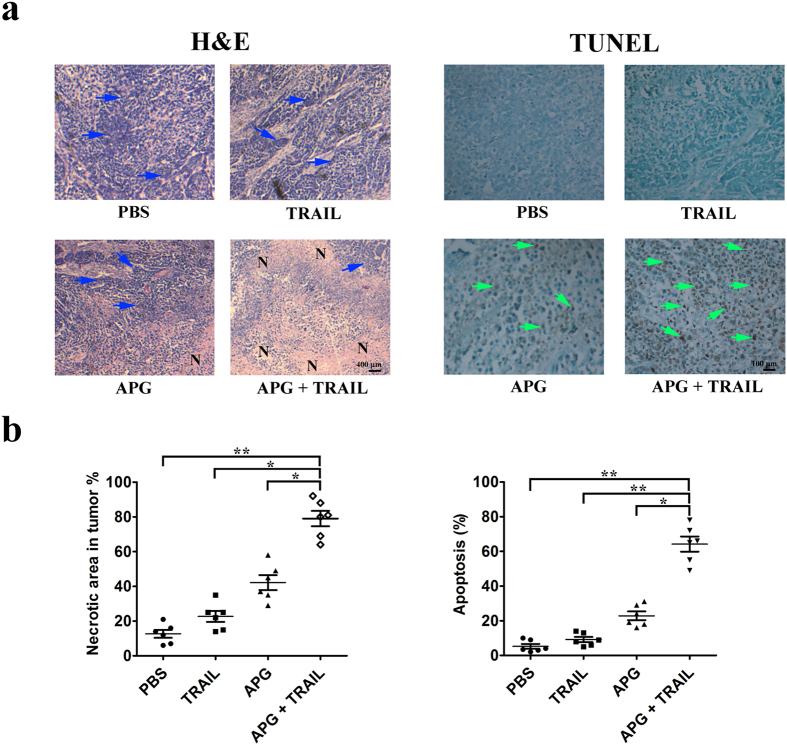
APG and TRAIL combined therapy induces necrosis of tumor and promotes tumor cell apoptosis. (**a**) Determination of tumor necrosis and apoptosis after combined treatment with APG and TRAIL. Tumor necrosis areas are shown by H&E staining and observed under light microscope (×100). The viable tumor cells are indicated by a *blue arrow*. TUNEL assay was used to detect apoptotic cells (original magnification, ×200). Positive cells for TUNEL staining are indicated by a *green arrow*. (**b**) Quota of tumor necrosis and apoptosis. Tumor necrosis was determined by software Image J. Two sections/mouse and three mice were prepared (mean ± SD, *P < 0.05, **p < 0.01). The ratio of apoptotic cells to total cells: TUNEL positive cells were counted from three fields of the highest density of positive-stained cells in each section to determine the percentage of apoptotic cells (mean ± SD, *p < 0.05, **p < 0.01).

Furthermore, light microscopy revealed that tumor tissues in mice receiving APG and TRAIL displayed more severe necrosis than control or APG or TRAIL single therapy (Fig. 8a). The percentage of necrotic area in tumors increased from 12.5% in mice receiving PBS, 22.7% in mice receiving TRAIL, 42.2% in mice receiving APG to 79.5% in mice receiving APG + TRAIL (Fig. 8b). APG or TRAIL single therapy or untreated control displayed tissue necrosis interspersed with viable tumor cells, whereas APG and TRAIL combined treatment induced large areas of continuous necrosis within tumors (Fig. 8a). TUNEL assay further suggested that the combined treatment of APG and TRAIL inhibited A549 tumors through the induction of programmed cell death *in vivo* (Fig. 8a,b).

## Discussion

**Figure 9:**
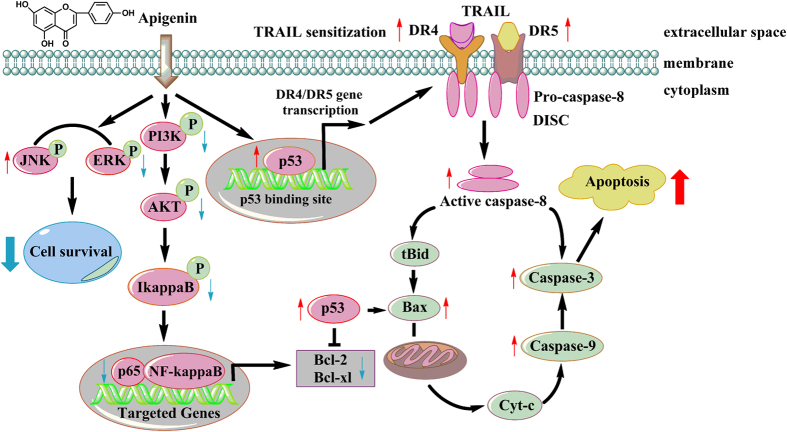
A working model for the synergistic effect of APG and TRAIL on NSCLC cells. APG treatment induces c-Jun N-terminal kinase (JNK) and subsequent c-Jun and p53 activation in NSCLC cells. The activation of p53 thus contributes to the upregulation of DR4 and DR5 levels. When NSCLC cells are treated with the combination of APG and TRAIL, TRAIL stimulates overexpressed DR4 and DR5, inducing the activation of caspase-8, which degrades Bid to t-Bid. Additionally, p53 accumulation accounts for the decrease in Bcl-2 level and also upregulates Bax level, contributing to Bax oligomerization. Furthermore, APG and TRAIL combined treatment inhibits AKT phosphorylation *via* the PI3K inhibition, which contributes to IκBα phosphorylation inhibition and degradation, suppresses the nuclear translocation of p65, and, in turn, decreases the expression of NF-κB target genes, such as c-FLIP, Bcl-2 and Bcl-xl. Thus, the increase in the Bax:Bcl-2 ratio induces the depolarization of the mitochondrial membrane with the release of cytochrome c and the consequent activation of caspase-9 and caspase-3, resulting in apoptosis of NSCLC cells. Moreover, the combination of APG and TRAIL also suppresses the activation of ERK pathway. All of the above finally sensitize NSCLC cells to TRAIL-induced apoptosis.

Combination therapies have been shown to produce a response rate that is higher than those obtained with single-agent chemotherapy in many types of cancer, including NSCLC. However, the success of therapies is limited, and one of the reasons for this limitation is drug resistance. Because only a small number of patients survive for more than a year after treatment, new agents and novel approaches are needed to improve the response to conventional therapies in NSCLC patients^28^. Understanding the molecular mechanism of apoptosis and the events that cause resistance to anticancer drugs is therefore crucial for developing new strategies for the therapeutic treatment of lung cancer, with the possibility of activating distinct or overlapping apoptotic pathways by combining different treatments to increase antitumor effects. In this study, we showed the synergistic interaction in lung cancer cells between TRAIL, a member of the tumor necrosis factor family currently under clinical trials, and APG, an edible plant-derived flavonoid. TRAIL can trigger rapid apoptosis of tumor cells while sparing normal cells, therefore representing a promising novel target for anti-tumor therapeutics. However, previous studies have shown that many cancer types are resistant to the apoptotic effects of TRAIL. Thus, it is important to develop agents that sensitize cancer cells to TRAIL-induced apoptosis to improve the therapeutic impact of TRAIL. Although APG has been tested as an antitumor agent against various human tumors, its antitumor activity is generally limited. Its potential application in combination with other anticancer drugs has not been thoroughly explored. Our results herein provided such an example that APG enhanced TRAIL-induced cell killing in lung cancer cells. Detailed studies demonstrated that APG downregulated Bcl-2, Bcl-xl and HSP70, and inhibited NF-κB, AKT and ERK prosurvival mediators. Targeting each of these pathways independently resulted in TRAIL-induced cell killing (Fig. 9). Furthermore, the synergistic interaction between APG and TRAIL in lung cancer cells was supported by the xenograft experiment in mice wherein coadministration of APG and TRAIL suppressed tumor growth more potently than APG or TRAIL alone. Therefore, combination therapeutic strategy using APG and TRAIL can be a powerful option for treatment of human lung cancer.

In the present study, we tested the *in vitro* and *in vivo* enhancing effect of APG on the cytotoxicity of TRAIL in a pair of NSCLC cell lines, A549 and H1299. Using MTT assay we found that APG inhibited the growth and proliferation of TRAIL-treated tumor cells synergistically. Compared to APG or TRAIL alone, low dosage of the two drugs in combination induced substantial apoptosis of tumor cells. The activation of caspase family members is essential for apoptosis through both death receptor as well as mitochondrial pathways. These caspases are classified into two groups, including initiator caspases (−2, −8, −9 and −10) and effector caspases (−3, −6 and −7), according to their functions. To further exploit the anti-tumor mechanism of APG and TRAIL, we detected the activation of caspase-8, caspase-9 and caspase-3. The data indicated that induced apoptosis of NSCLC cells by the combination of APG and TRAIL was mediated through caspase-8/caspase-9/caspase-3 activation. In turn, caspases activation resulted in PARP cleavage, nuclear condensation, and eventually, the induction of apoptosis. Caspase-8 mediates cleavage of Bid to tBid and the translocation of tBid to mitochondria provides a link between extrinsic and intrinsic pathways of apoptosis^29^. Increase in Bax:Bcl-2 ratio is a key factor to induce the release of several pro-apoptotic molecules from mitochondria^30^. Upregulation of various anti-apoptotic molecules, such as Bcl-2 and Bcl-xl, protects the cancer cells from induction of apoptosis^31^. In the current study, we found that APG and TRAIL combined treatment could cause maximum upregulation of Bax and Bad with a concomitant downregulation of Bcl-2 and Bcl-xl so as to cause an increase in the Bax:Bcl-2 ratio for triggering activation of the caspases related to the mitochondrial cascade for apoptotic death in A549 and H1299 cells.

The NF-κB pathway is one of the most important cellular signal transduction pathways involved in immunity, inflammation, proliferation, and in defense against apoptosis^32^. Activation of NF-κB is a frequent event in cancer cells, pointing toward that it could be an attractive therapeutic target during the treatment^33^,^34^. In cells, the p65-p50 heterodimer is maintained in an inactive state due to binding of IκB. However, when IκB is phosphorylated *via* IκB kinase, phospho-IκB is detached from p65-p50 dimer to be degraded. Free p65-p50 heterodimer then can enter the nucleus, and bind to specific DNA sequences to induce the transcription of target genes related to tumor promotion, cell survival signaling, and inflammation^35^. In this study, we found that APG monotherapy or APG and TRAIL combined treatment abrogated the phosphorylation of IκBα and nuclear translocation of p65. This finding is consistent with that of other reports^36^,^37^. NF-κB is generally considered to be a survival factor that activates expression of various anti-apoptotic genes, such as Bcl-xl, Bcl-2, and c-FLIP that block apoptosis^38^. Inhibition of NF-κB will lead to downregulation of the NF-κB-regulated anti-apoptotic proteins, thereby promoting apoptotic cell death. Indeed, combined treatment with APG and TRAIL significantly decreased the expression levels of Bcl-2, Bcl-xl and c-FLIP, and finally resulted in cell apoptosis. Thus, the suppression of NF-κB by APG could provide specific and effective way to sensitize NSCLC cells to TRAIL-induced apoptosis.

The serine/threonine kinase protein kinase B or AKT (PKB/AKT), a downstream target of phosphoinositide 3-kinase (PI3K), plays critical regulatory roles in cell signaling, affecting such divergent cellular processes as apoptosis, cellular proliferation, and metabolism^39^,^40^. AKT is also activated in various cancer cells, and thus AKT signaling has become a target for cancer chemotherapy. Several reports have suggested that APG exerts its anticancer effects by blocking the AKT pathway^41^,^42^. In the present study, APG combined with TRAIL decreased both the expression and phosphorylation level of AKT and PI3K in an APG-dose dependent manner. Thus, the inhibition of the PI3K/AKT pathway may be an important mechanism underlying the effects of APG combined with TRAIL in human lung cancer A549 and H1299 cells. It has been reported that AKT can promote NF-κB activity^43^. Thus, the deactivation of AKT may lead to transcriptional inhibition of NF-κB, and the previously well-characterized downregulation of Bcl-2 and Bcl-xl expression by inactivated NF-κB. In addition, co-treatment with APG and TRAIL also led to JNK activation. JNK is activated by many cellular stresses, including oxidative stresses, and DNA-damaging agents^22^ and plays important roles in cell proliferation/survival and apoptosis^44^. Recent studies have indicated that one of the anti-apoptotic functions of NF-κB is to downregulate JNK activation^45^. Therefore, we demonstrated that the combined treatment with APG and TRAIL led to the activation of JNK in NSCLCs concurrent to the downregulation of NF-κB, thus contributing to apoptosis. Thereby, the suppression of AKT, inhibition of NF-κB, upregulation of JNK activity and subsequent reduction of Bcl-2 and Bcl-xl expression might contribute to the increased sensitivity of NSCLC cells to APG and TRAIL-mediated apoptosis (Fig. 9).

The p53 tumor suppressor inhibits cellular proliferation by inducing cell cycle arrest and apoptosis in response to cellular stresses including DNA damage, growth factor deprivation, hypoxia, and oncogene activation^46^,^47^. It has been reported that JNK could upregulate pro-apoptotic genes and downregulate anti-apoptotic genes through the transactivation of several transcription factors, including c-JUN and p53^44^. Our western blotting data also indicated that APG at the concentration of 20 μM or 40 μM significantly increased the expression of p53, and the previously well-characterized upregulation of DR4 and DR5 levels by activated p53^48^,^49^. Therefore, APG treatment could upregulate DR4 and DR5 expression in NSCLC cells through activating p53, thus contributing to TRAIL-induced apoptosis. To detect whether p53 is the key mediator of apoptosis produced by the combination of APG and TRAIL, A549 cells were pretreated with the p53 inhibitor PFT-α. The results indicated that PFT-α not only reversed the synergistic apoptotic effect of the combination of APG and TRAIL, but also blocked the accompanying changes in p53 and DR5 expression levels. These data clearly indicate that p53 plays an important role in the synergistic apoptotic effect.

Heat shock proteins (HSPs) are molecular chaperones that transport and stabilize proteins within the cell. Many human tumor cells display heightened levels of HSP27, HSP70, and/or HSP90, suggesting an association of cellular transformation with altered stress protein levels^50^. Previous studies found that suppression of HSP70 or HSP27 expression could sensitize NSCLC cells to TRAIL-induced apoptosis by upregulating DR4 and DR5^27^,^51^. Herein, we found that 40 μM APG combined with TRAIL significantly reduced HSP70 protein level without exhibiting any effects on the expression of HSP27 and HSP90. Thus, the downregulation of HSP70 expression might also contribute to the increased sensitivity of NSCLC cells to TRAIL-mediated apoptosis.

Our experiments using a tumor xenograft model showed that APG-induced sensitization to TRAIL also occurred *in vivo* and this combination therapy efficiently inhibited tumor growth. Our findings argue that the novel anticancer effects of APG in lung cancer cells involve TRAIL sensitization through the augmented expression of its receptors *via* NF-κB downregulation and deactivation, JNK activation and the subsequent increase of p53. Wherefore it is suggested that APG could be useful as a new strategy for overcoming TRAIL resistance in cancer therapy.

In conclusion, we provided evidence that APG alone showed limited inhibition potency on the growth of human lung cancer cell lines *in vitro*. However, at a low concentration, APG synergistically induced cell apoptosis through multiple targets including caspases and NF-κB pathways in NSCLC cell lines when combined with TRAIL. Furthermore, combined treatment with the two drugs effectively reduced growth of xenografted A549 cells grown in athymic mice without exhibiting any toxicity for the animals. Based on the synergistic antitumor activity profiles of combined APG and TRAIL treatments *in vitro* and *in vivo* and the absence of cytotoxicity in normal tissues, we believe that APG has strong therapeutic value for use in combination with TRAIL against lung cancer that warrants further investigation.

## Materials and Methods

### Cells, cell culture, and reagents

NSCLC cell lines A549 and H1299, and human embryonic renal HEK293 cells were purchased from the American Type Culture Collection (ATCC, Philadelphia, PA, USA). HEK293 cells were grown in Dulbecco’s modified Eagle’s medium (DMEM) (HyClone, Logan, UT, USA) supplemented with 10% (v/v) fetal bovine serum (HyClone, Logan, UT, USA) and 1% penicillin-streptomycin (Invitrogen, Carlsbad, CA, USA). A549 and H1299 cells were grown in RPMI 1640 (HyClone, Logan, UT, USA) supplemented with 10% (v/v) fetal bovine serum (HyClone, Logan, UT, USA) and 1% penicillin-streptomycin (Invitrogen, Carlsbad, CA, USA). All cells were cultured in a humidified CO_2_ incubator at 37 °C. MEK inhibitor AZD6244 (Calbiochem, Merck Biosciences, Darmstadt, Germany), PI3K/AKT inhibitor LY294002, p53 inhibitor Pifithrin-α (PFT-α), and HSP70 inhibitor Pifithrin-μ (PFT-μ; all from Sigma-Aldrich, St. Louis, MO, USA) were dissolved in dimethyl sulfoxide and freshly prepared each time before use. The working concentration for AZD6244, LY294002, PFT-α and PFT-μ was 2 μM, 5 μM, 20 μM and 5 μM respectively. 4,6-diamidino-2-phenylindole (DAPI) was purchased from Sigma-Aldrich, St. Louis, MO, USA.

### Cell proliferation assay

The effects of TRAIL, APG or the combination of two drugs on cell proliferation were assessed by the MTT assay. Cells in the exponential growth phase were seeded into a 96-well plate at a density of 5000 cells per well. After 24 h, APG (0–160 μM), TRAIL (25 ng/mL), or the combination of both substances was added to the medium. The cells were incubated at 37 °C for 24 h, then the cell viability was determined by the colorimetric MTT [3-(4, 5-dimethylthiazol-2-yl)-2, 5-diphenyl-2H-tetrazolium bromide] assay at 570 nm by TECAN Safire Fluorescence Absorbance and Luminescence Reader (Vienna, VA, USA). The cell viability was calculated according to the formula: Cell viability (%) = average A_570 nm_ of treated group/average A_570 nm_ of control group × 100%. Each experiment was performed in quadruplicate and repeated at least three times.

### Annexin V-fluorescein isothiocyanate/propidium iodide assay

To quantify the percentage of cells undergoing apoptosis, we used the Annexin V-FITC kit as described by the manufacturer (BD Biosciences, CA, USA). Briefly, A549, H1299 and HEK293 cells were incubated for 18 hours with TRAIL (25 ng/mL), or with the indicated concentration of APG alone or with the combination of both substances. Next, the treated cells were collected and trypsinized for 3–5 min. The digested cells were washed twice with cold phosphate buffered saline (PBS) and resuspended in binding buffer at a concentration of 1 × 10^6^ cells/mL. After incubation, 100 μL of the solution was transferred to a 5 mL culture tube, and 5 μL of Annexin V-FITC (20 μg/mL) and 5 μL of PI (20 μg/mL) were added. The tube was gently centrifuged at 1000 rpm for 5 min and incubated for 15 min at room temperature in the dark. At the end of incubation, 400 μL of binding buffer was added, and the cells were analyzed immediately by flow cytometry (BD Biosciences, CA, USA). Flow cytometry analysis was performed with untreated A549, H1299 and HEK293 cells as control.

### Colony-forming assay

Colony-forming assay was performed as previously described^51^. Briefly, About 300 cells in log phase were plated into 60 mm tissue culture Petri-dish (Greiner) in triplicate with 3 mL of culture medium and grown at 37 °C with 5% CO_2_. After 48 h culture for cell adherence to the plate, rinsed with fresh medium, APG (20 μM), TRAIL (25 ng/mL) or APG + TRAIL were added to the medium. 48 h later, the cells were washed twice with PBS and then incubated in drug-free medium. The medium was changed every 5 days. After culturing for additional 10–14 days, the medium was discarded and each dish was washed twice with PBS carefully. The cells were fixed with methanol for 15 min and stained with a 1:10 dilution of Giemsa regent (Merck Biosciences, Darmstadt, Germany) for 10 min. Any grouping of cells containing 30 or more cells was counted as a colony using an inverted microscope (Zeiss, 40-fold magnification). Colony numbers were determined from triplicate plates. Colony growth was related to the control value without any treatment. The sensitivity of APG-treated (A1) and non-treated (A2) cells to TRAIL treatment was calculated using the following formulas:


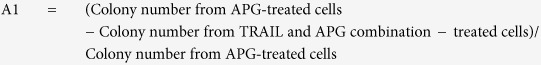



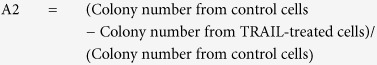


The sensitization ratio of cells to TRAIL was calculated as A1/A2.

### Caspase activity assay

Caspase-3 and caspase-9 activities were measured using colorimetric activity assay kits (Chemicon International, CA, USA) following the manufacturer’s instructions. The assay is based on the cleavage of the chromogenic substrates, DEVD-pNA and LEHD-pNA, by caspase-3 and caspase-9, respectively. Cells were lysed in chilled lysis buffer on ice for 10 min and centrifuged for 5 min at 10,000× *g*. Caspase substrate solution containing the specific peptide substrate was then added to the supernatant and incubated for 2 h at 37 °C before measurement by ELISA reader at 405 nm.

### Western blot analysis

Cells were incubated with TRAIL (25 ng/mL) and the indicated concentration of APG alone or in combination for 24 h, and then lysed with RIPA buffer (Beyotime, China) with protease inhibitor cocktail tablets (Complete Mini, EDTA free; Roche, Basel, Switzerland). Supernatants were collected by centrifugation at 12,000× *g* for 10 min and protein concentration was determined by the Bio-Rad protein assay method (Bio-Rad, Hercules, CA). In addition, nuclear extracts were prepared as described by Schreiber *et al*.^52^. Western blotting used standard protocols. Proteins were separated by SDS-PAGE and transferred onto nitrocellulose membranes that were blocked with 5% non-fat milk in TBS containing 0.1% Tween-20, and incubated with primary antibodies: caspase-8, cleaved caspase-8, cleaved caspase-3, caspase-9, p-JNK, c-JUN, p-c-JUN, AKT, p-AKT (Ser 473), p53 (Cell Signaling Technology, Beverly, MA, USA), IкB-α, p-IкB-α (Sigma, St. Louis, MO, USA), NF-κB p65 (Invitrogen, Carlsbad, CA, USA), α-tubulin, GAPDH, Lamin B, ERK, p-ERK, P38, p-P38, DR4, DR5, poly (ADP-ribose) polymerase (PARP), Bad, Bax, Bcl-xl, Bcl-2 (Santa Cruz Biotechnology, Santa Cruz, CA, USA). Secondary antibodies were coupled to horseradish peroxidase, and were goat anti-rabbit or goat anti-mouse (Cell Signaling Technology, Beverly, MA, USA). Bound antibodies were then visualized with ECL plus Western blotting detection reagents (GE Healthcare). Signal intensity was quantified by densitometry using the software Image J (NIH, Bethesda, MD). All experiments were done in triplicate and performed at least three times independently.

### RNA extraction and quantitative real-time PCR

Cells were incubated with TRAIL (25 ng/mL), APG (20 μM) or the combination of two drugs for 24 h. Total RNA was then extracted from treated cells using a TRIzol reagent (Invitrogen, Carlsbad, CA, USA) following the manufacturer’s instructions and was used to prepare cDNA by PrimeScript RT reagent Kit (Takara, Otsu, Shiga, Japan). Quantitative real-time PCR was performed with SsoFast EvaGreen Supermix on a CFX96 Real-Time System (Bio-Rad Laboratories, Hercules, CA, USA). The sequences of PCR primers used in our study were synthesized commercially, and are shown as follows: DR4 upstream: 5′-CCGCGGCCACACCCAGCAAAGT-3′; DR4 downstream: 5′-GCAGCAGGACCCCGACGACGACA-3′; DR5 upstream: 5′-GTGGGGACCCTGAGCGTGTG-3′; DR5 downstream: 5′-CTCCAAGGCATCCAGCAGGGTGTG-3′. c-FLIP upstream: 5′-CCTCCGCACATCCGTGAA-3′; c-FLIP downstream: 5′-AGGTCTCTTGAAGATATTTTGTGTCGTT-3′. Bcl-2 upstream: 5′-AACATCGCCCTGTGGATGAC-3′; Bcl-2 downstream: 5′-GGCCGTACAGTTCCACAAAG-3′. Bcl-xl upstream: 5′-GGCCACTTACCTGAATGACC-3′; Bcl-xl downstream: 5′-AAGAGTGAGCCCAGCAGAAC-3′. The glyceraldehydes 3-phosphatase dehydrogenase (GAPDH) gene was used as the reference gene. GAPDH upstream: 5′-AACGACCCCTTCATTGAC-3′; GAPDH downstream: 5′-TCCACGACATACTCAGCAC-3′. The thermal dissociation curve of all the primers pairs are shown in Supplementary Fig. S8 in comparison with the predicted dissociation curves from uMelt (https://dna.utah.edu/umelt/umelt.html) for the same primers pairs. All data were means of fold change of triplicate analysis and normalized with those of GAPDH.

### DAPI staining

DAPI staining was applied for morphological assessment of nuclei. NSCLC ells were incubated with TRAIL (25 ng/mL), APG (20 μM) or the combination of two drugs for 18 h. Treated A549 or H1299 cells in 6-well plate were rinsed twice with cold PBS and stained with DAPI solution (1 μg/mL) for 10 min at 37 °C in dark room. Stained cells were washed twice with cold PBS. Finally, an inverted fluorescence microscopy (CKX41, OLYMPUS, Japan) was used to photograph the cells in the plate. At least 200 cells were counted and classified according to the condensed nuclei.

### Animals

Athymic nude mice (6–8 weeks of age) were obtained from Shanghai Laboratory Animal Center (Shanghai, China) and housed under germfree conditions. All animals received human care according to Chinese legal requirements. The experiments were approved by Nanjing University Animal Care and Use Committee (20150153), and we strictly followed these rules during our experiments.

### *In vivo* animal tumor model experiment

A549 cells (5 × 10^5^ cells in 30 μL) were injected subcutaneously into the dorsal flanks of mice. Tumor volume was monitored by measuring the two maximum perpendicular tumor diameters with callipers every other day. All tumor-bearing mice were divided randomly into 4 groups, and treatment was initiated on the 8^th^ day when the volume of tumor reached a size of approximately 50 mm^3^. The mice were injected intraperitoneally (i.p.) with APG (10 μg/injection/mouse), TRAIL (100 μg/injection/mouse) or the combination of two drugs every two day for a total of three weeks. Control mice received i.p. injection of PBS. Antitumor activity of treatments was evaluated by tumor growth inhibition. The formula, tumor volume = length × width^2^ × 0.52 was used to mimic the tumor volume. At the end of study, the tumors were collected and weighed. The body weight and liver mass were also examined to evaluate the toxicity of different treatments *in vivo*.

In a parallel animal assay (totally 4 groups, and 3 mice per group), the tumor establishment and drug treatment are the same as described above. On the 21^th^ day, mice were euthanized. Tumors and livers were collected, fixed with 4% formaldehyde, embedded in paraffin and sectioned for haematoxylin and eosin (H&E) staining according to standard histological procedures. Apoptotic cells in tumor sections (two sections per mouse, three mice in total) were visualized by the TUNEL technique according to the manufacturer’s instruction (Merck Biosciences, Darmstadt, Germany).

### Calculation of tumor doubling time and combination index

The tumor doubling time (TDT) and combination index (CI) were calculated using GraphPad Prism v 5.0. TDT values were generated from exponential growth curves, which had been fitted to % change in tumor volume data (*r*^2^ > 0.70). Our CI calculations were adapted^53^ to apply to TDT values. First, the TDT value for untreated mice was subtracted from the TDT value for each treatment group to obtain ‘blanked’ TDT values (TDT_B_). Then, the CI was calculated as the ratio of TDT_B_ values of combination treatment to individual treatments: CI = (TDT_B_ combination of APG and TRAIL)/(TDT_B_ APG alone + TDT_B_ TRAIL alone).

### Statistical analysis

Statistical analysis was carried out using the SPSS software (version 11.0; SPSS, Chicago, IL). Data were expressed as the mean ± standard deviations (SD). For paired data, statistical analyses were performed using two-tailed Student’s t-tests. For multiple comparisons, statistical analyses were performed using one-way analysis of variance (ANOVA) with a Tukey post-test. For all analyses, *P* < 0.05 was considered statistically significant.

## Additional Information

**How to cite this article**: Chen, M. *et al*. Apigenin potentiates TRAIL therapy of non-small cell lung cancer *via* upregulating DR4/DR5 expression in a p53-dependent manner. *Sci. Rep.* **6**, 35468; doi: 10.1038/srep35468 (2016).

## Supplementary Material

Supplementary Information

## References

[b1] Malvezzi M. . European cancer mortality predictions for the year 2015: does lung cancer have the highest death rate in EU women? Annals of oncology: official journal of the European Society for Medical Oncology/ESMO 26, 779–786, 10.1093/annonc/mdv001 (2015). 25623049

[b2] Siegel R. L., Miller K. D. & Jemal A. Cancer statistics, 2015. CA: a cancer journal for clinicians 65, 5–29, 10.3322/caac.21254 (2015). 25559415

[b3] Nawaz K. & Webster R. M. The non-small-cell lung cancer drug market. Nature reviews. Drug discovery 15, 229–230, 10.1038/nrd.2016.42 (2016). 27032828

[b4] Steinke K. Lung tumors. Recent results in cancer research. Fortschritte der Krebsforschung. Progres dans les recherches sur le cancer 167, 107–122 (2006). 17044300 10.1007/3-540-28137-1_8

[b5] Chang D. W. . c-FLIP(L) is a dual function regulator for caspase-8 activation and CD95-mediated apoptosis. The EMBO journal 21, 3704–3714, 10.1093/emboj/cdf356 (2002). 12110583 PMC125398

[b6] Wiley S. R. . Identification and characterization of a new member of the TNF family that induces apoptosis. Immunity 3, 673–682 (1995). 8777713 10.1016/1074-7613(95)90057-8

[b7] Duiker E. W. . The clinical trail of TRAIL. European journal of cancer 42, 2233–2240, 10.1016/j.ejca.2006.03.018 (2006). 16884904

[b8] Dyer M. J., MacFarlane M. & Cohen G. M. Barriers to effective TRAIL-targeted therapy of malignancy. Journal of clinical oncology: official journal of the American Society of Clinical Oncology 25, 4505–4506, 10.1200/JCO.2007.13.1011 (2007). 17906217

[b9] Mahalingam D., Szegezdi E., Keane M., de Jong S. & Samali A. TRAIL receptor signalling and modulation: Are we on the right TRAIL? Cancer treatment reviews 35, 280–288, 10.1016/j.ctrv.2008.11.006 (2009). 19117685

[b10] Cai J., Zhao X. L., Liu A. W., Nian H. & Zhang S. H. Apigenin inhibits hepatoma cell growth through alteration of gene expression patterns. Phytomedicine: international journal of phytotherapy and phytopharmacology 18, 366–373, 10.1016/j.phymed.2010.08.006 (2011). 20850954

[b11] Khan T. H. & Sultana S. Apigenin induces apoptosis in Hep G2 cells: possible role of TNF-alpha and IFN-gamma. Toxicology 217, 206–212, 10.1016/j.tox.2005.09.019 (2006). 16289292

[b12] Shukla S. & Gupta S. Molecular targets for apigenin-induced cell cycle arrest and apoptosis in prostate cancer cell xenograft. Molecular cancer therapeutics 5, 843–852, 10.1158/1535-7163.MCT-05-0370 (2006). 16648554

[b13] Shukla S. . Blockade of beta-catenin signaling by plant flavonoid apigenin suppresses prostate carcinogenesis in TRAMP mice. Cancer research 67, 6925–6935, 10.1158/0008-5472.CAN-07-0717 (2007). 17638904

[b14] Nicholas C. . Apigenin blocks lipopolysaccharide-induced lethality *in vivo* and proinflammatory cytokines expression by inactivating NF-kappaB through the suppression of p65 phosphorylation. Journal of immunology 179, 7121–7127 (2007). 10.4049/jimmunol.179.10.7121 17982104

[b15] Lee S. H. . Enhanced anti-tumor effect of combination therapy with gemcitabine and apigenin in pancreatic cancer. Cancer letters 259, 39–49, 10.1016/j.canlet.2007.09.015 (2008). 17967505

[b16] Crespo I. . Differential effects of dietary flavonoids on reactive oxygen and nitrogen species generation and changes in antioxidant enzyme expression induced by proinflammatory cytokines in Chang Liver cells. Food and chemical toxicology: an international journal published for the British Industrial Biological Research Association 46, 1555–1569, 10.1016/j.fct.2007.12.014 (2008). 18234413

[b17] Chan L. P. . Apigenin induces apoptosis via tumor necrosis factor receptor- and Bcl-2-mediated pathway and enhances susceptibility of head and neck squamous cell carcinoma to 5-fluorouracil and cisplatin. Biochimica et biophysica acta 1820, 1081–1091, 10.1016/j.bbagen.2012.04.013 (2012). 22554915

[b18] Daniel B. & DeCoster M. A. Quantification of sPLA2-induced early and late apoptosis changes in neuronal cell cultures using combined TUNEL and DAPI staining. Brain research. Brain research protocols 13, 144–150, 10.1016/j.brainresprot.2004.04.001 (2004). 15296851

[b19] Jost P. J. & Ruland J. Aberrant NF-kappaB signaling in lymphoma: mechanisms, consequences, and therapeutic implications. Blood 109, 2700–2707, 10.1182/blood-2006-07-025809 (2007). 17119127

[b20] Dan H. C. . Akt-dependent regulation of NF-{kappa}B is controlled by mTOR and Raptor in association with IKK. Genes & development 22, 1490–1500, 10.1101/gad.1662308 (2008). 18519641 PMC2418585

[b21] Budhraja A. . Apigenin induces apoptosis in human leukemia cells and exhibits anti-leukemic activity *in vivo*. Molecular cancer therapeutics 11, 132–142, 10.1158/1535-7163.MCT-11-0343 (2012). 22084167 PMC4430727

[b22] Ouyang D. Y., Wang Y. Y. & Zheng Y. T. Activation of c-Jun N-terminal kinases by ribotoxic stresses. Cellular & molecular immunology 2, 419–425 (2005). 16426491

[b23] Xing Y. X., Li P., Miao Y. X., Du W. & Wang C. B. Involvement of ROS/ASMase/JNK signalling pathway in inhibiting UVA-induced apoptosis of HaCaT cells by polypeptide from Chlamys farreri. Free radical research 42, 12–19, 10.1080/10715760701762415 (2008). 18324519

[b24] LeBlanc H. N. & Ashkenazi A. Apo2L/TRAIL and its death and decoy receptors. Cell death and differentiation 10, 66–75, 10.1038/sj.cdd.4401187 (2003). 12655296

[b25] Tomasetti M. . Alpha-tocopheryl succinate induces DR4 and DR5 expression by a p53-dependent route: implication for sensitisation of resistant cancer cells to TRAIL apoptosis. FEBS letters 580, 1925–1931, 10.1016/j.febslet.2006.02.054 (2006). 16529749

[b26] Davies B. R. . AZD6244 (ARRY-142886), a potent inhibitor of mitogen-activated protein kinase/extracellular signal-regulated kinase kinase 1/2 kinases: mechanism of action *in vivo*, pharmacokinetic/pharmacodynamic relationship, and potential for combination in preclinical models. Molecular cancer therapeutics 6, 2209–2219, 10.1158/1535-7163.MCT-07-0231 (2007). 17699718

[b27] Zhuang H. . Suppression of HSP70 expression sensitizes NSCLC cell lines to TRAIL-induced apoptosis by upregulating DR4 and DR5 and downregulating c-FLIP-L expressions. Journal of molecular medicine 91, 219–235, 10.1007/s00109-012-0947-3 (2013). 22948392

[b28] Peto R. . Mortality from smoking worldwide. British medical bulletin 52, 12–21 (1996). 8746293 10.1093/oxfordjournals.bmb.a011519

[b29] Fulda S. & Debatin K. M. Extrinsic versus intrinsic apoptosis pathways in anticancer chemotherapy. Oncogene 25, 4798–4811, 10.1038/sj.onc.1209608 (2006). 16892092

[b30] Karmakar S., Banik N. L., Patel S. J. & Ray S. K. Curcumin activated both receptor-mediated and mitochondria-mediated proteolytic pathways for apoptosis in human glioblastoma T98G cells. Neuroscience letters 407, 53–58, 10.1016/j.neulet.2006.08.013 (2006). 16949208

[b31] George J., Banik N. L. & Ray S. K. Bcl-2 siRNA augments taxol mediated apoptotic death in human glioblastoma U138MG and U251MG cells. Neurochemical research 34, 66–78, 10.1007/s11064-008-9659-z (2009). 18357521 PMC11926550

[b32] Dutta J., Fan Y., Gupta N., Fan G. & Gelinas C. Current insights into the regulation of programmed cell death by NF-kappaB. Oncogene 25, 6800–6816, 10.1038/sj.onc.1209938 (2006). 17072329

[b33] Chen P. H. & Yang C. R. Decoy receptor 3 expression in AsPC-1 human pancreatic adenocarcinoma cells via the phosphatidylinositol 3-kinase-, Akt-, and NF-kappa B-dependent pathway. Journal of immunology 181, 8441–8449 (2008). 10.4049/jimmunol.181.12.8441 19050262

[b34] Fujioka S. . Inhibition of constitutive NF-kappa B activity by I kappa B alpha M suppresses tumorigenesis. Oncogene 22, 1365–1370, 10.1038/sj.onc.1206323 (2003). 12618762

[b35] He G. & Karin M. NF-kappaB and STAT3 - key players in liver inflammation and cancer. Cell research 21, 159–168, 10.1038/cr.2010.183 (2011). 21187858 PMC3193410

[b36] Seo H. S. . Apigenin induces apoptosis via extrinsic pathway, inducing p53 and inhibiting STAT3 and NFkappaB signaling in HER2-overexpressing breast cancer cells. Molecular and cellular biochemistry 366, 319–334, 10.1007/s11010-012-1310-2 (2012). 22527937

[b37] Wu D. G. . Apigenin potentiates the growth inhibitory effects by IKK-beta-mediated NF-kappaB activation in pancreatic cancer cells. Toxicology letters 224, 157–164, 10.1016/j.toxlet.2013.10.007 (2014). 24148603

[b38] Li-Weber M. Targeting apoptosis pathways in cancer by Chinese medicine. Cancer letters 332, 304–312, 10.1016/j.canlet.2010.07.015 (2013). 20685036

[b39] Carnero A., Blanco-Aparicio C., Renner O., Link W. & Leal J. F. The PTEN/PI3K/AKT signalling pathway in cancer, therapeutic implications. Current cancer drug targets 8, 187–198 (2008). 18473732 10.2174/156800908784293659

[b40] West K. A., Castillo S. S. & Dennis P. A. Activation of the PI3K/Akt pathway and chemotherapeutic resistance. Drug resistance updates: reviews and commentaries in antimicrobial and anticancer chemotherapy 5, 234–248 (2002). 12531180 10.1016/s1368-7646(02)00120-6

[b41] Lee W. J., Chen W. K., Wang C. J., Lin W. L. & Tseng T. H. Apigenin inhibits HGF-promoted invasive growth and metastasis involving blocking PI3K/Akt pathway and beta 4 integrin function in MDA-MB-231 breast cancer cells. Toxicology and applied pharmacology 226, 178–191, 10.1016/j.taap.2007.09.013 (2008). 17961621

[b42] Long X., Fan M., Bigsby R. M. & Nephew K. P. Apigenin inhibits antiestrogen-resistant breast cancer cell growth through estrogen receptor-alpha-dependent and estrogen receptor-alpha-independent mechanisms. Molecular cancer therapeutics 7, 2096–2108, 10.1158/1535-7163.MCT-07-2350 (2008). 18645020 PMC2559959

[b43] Rogers R. . Cross-talk between the Akt and NF-kappaB signaling pathways inhibits MEHP-induced germ cell apoptosis. Toxicological sciences: an official journal of the Society of Toxicology 106, 497–508, 10.1093/toxsci/kfn186 (2008). 18755736 PMC2581679

[b44] Dhanasekaran D. N. & Reddy E. P. JNK signaling in apoptosis. Oncogene 27, 6245–6251, 10.1038/onc.2008.301 (2008). 18931691 PMC3063296

[b45] Papa S., Zazzeroni F., Pham C. G., Bubici C. & Franzoso G. Linking JNK signaling to NF-kappaB: a key to survival. Journal of cell science 117, 5197–5208, 10.1242/jcs.01483 (2004). 15483317

[b46] Schuler M. & Green D. R. Mechanisms of p53-dependent apoptosis. Biochemical Society transactions 29, 684–688 (2001). 11709054 10.1042/0300-5127:0290684

[b47] Shen Y. & White E. p53-dependent apoptosis pathways. Advances in cancer research 82, 55–84 (2001). 11447765 10.1016/s0065-230x(01)82002-9

[b48] Liu X., Yue P., Khuri F. R. & Sun S. Y. p53 upregulates death receptor 4 expression through an intronic p53 binding site. Cancer research 64, 5078–5083, 10.1158/0008-5472.CAN-04-1195 (2004). 15289308

[b49] Sheikh M. S. . p53-dependent and -independent regulation of the death receptor KILLER/DR5 gene expression in response to genotoxic stress and tumor necrosis factor alpha. Cancer research 58, 1593–1598 (1998). 9563466

[b50] Jaattela M. Escaping cell death: survival proteins in cancer. Experimental cell research 248, 30–43, 10.1006/excr.1999.4455 (1999). 10094811

[b51] Zhuang H. . Down-regulation of HSP27 sensitizes TRAIL-resistant tumor cell to TRAIL-induced apoptosis. Lung cancer 68, 27–38, 10.1016/j.lungcan.2009.05.014 (2010). 19540014

[b52] Schreiber E., Matthias P., Muller M. M. & Schaffner W. Rapid detection of octamer binding proteins with ‘mini-extracts’, prepared from a small number of cells. Nucleic acids research 17, 6419 (1989). 2771659 10.1093/nar/17.15.6419 PMC318318

[b53] Chou T. C. Theoretical basis, experimental design, and computerized simulation of synergism and antagonism in drug combination studies. Pharmacological reviews 58, 621–681, 10.1124/pr.58.3.10 (2006). 16968952

