# Supplementary material for: Apigenin potentiates TRAIL therapy of non-small cell lung cancer via upregulating DR4/DR5 expression in a p53-dependent manner
Source: Sci Rep. 2016 Oct 18;6:35468. doi: 10.1038/srep35468 (PMC5067669; doi:10.1038/srep35468)
Supplement: Supplementary Information [file srep35468-s1.pdf]

Apigenin potentiates TRAIL therapy of non-small cell lung cancer *via* upregulating DR4/DR5 expression in a p53-dependent manner

Minghui Chen[1,2], Xueshi Wang[1], Daolong Zha[1], Fangfang Cai[1], Wenjing Zhang[2], Yan He[2], Qilai Huang[2,3], Hongqin Zhuang[1,3,*], Zi-Chun Hua[1,2,4,*]

HEK293

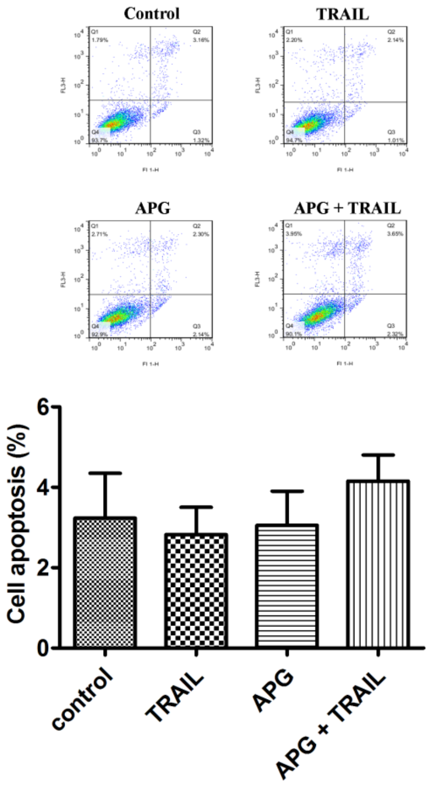

Supplementary Figure S1. APG and TRAIL combined treatment has no effect on the apoptotic rate of HEK293 cells. HEK293 cells were exposed to APG (20 μM) and/or TRAIL (25 ng/mL). Eighteen hours later, all cells were harvested for flow cytometry analysis. Annexin V/PI-stained cells were analyzed and the percentage of apoptotic cells was determined. The results shown are representative of three different experiments. Data are represented as mean ± SD.

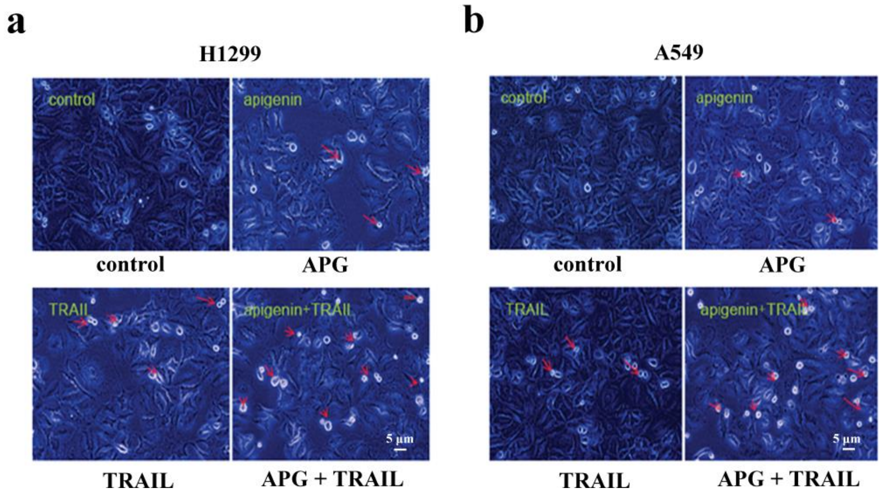

Supplementary Figure S2. APG enhances TRAIL-induced apoptosis of NSCLC cells. DAPI staining in treated groups in H1299 (**a**) and A549 (**b**) cells: apoptotic cells show condensed nuclei with karyorrhexis which are stained bright blue (Arrows).

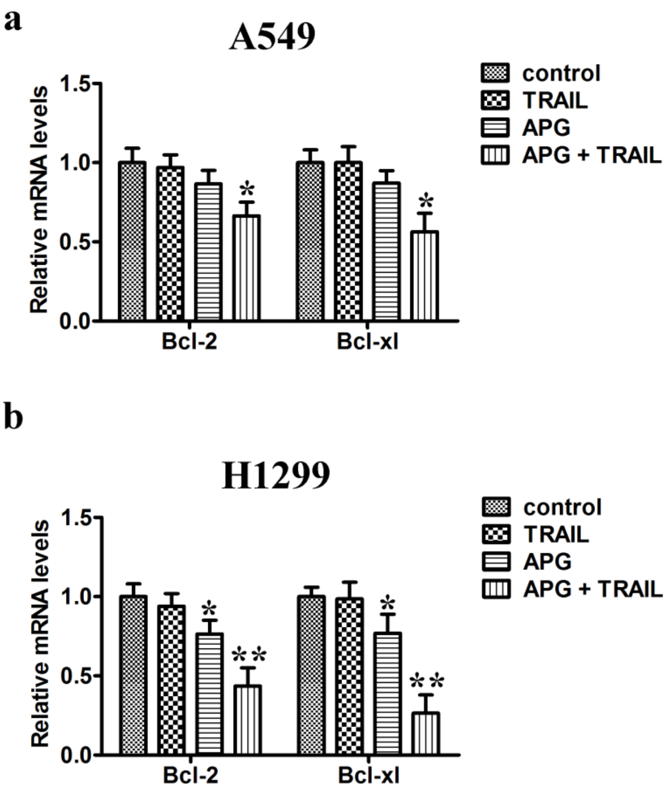

Supplementary Figure S3. Effect of APG and TRAIL on Bcl-2 and Bcl-xl mRNA levels. A549 cells (**a**) and H1299 cells (**b**) were treated with APG (20 μM) in the absence or the presence of TRAIL (25 ng/mL) for 24 h. Q-PCR analysis was performed to detect the level of the mRNA transcripts of Bcl-2 and Bcl-xl. The results shown are representative of three independent experiments. The histogram shows the mean ± SD. **p* < 0.05, ***p* < 0.01.

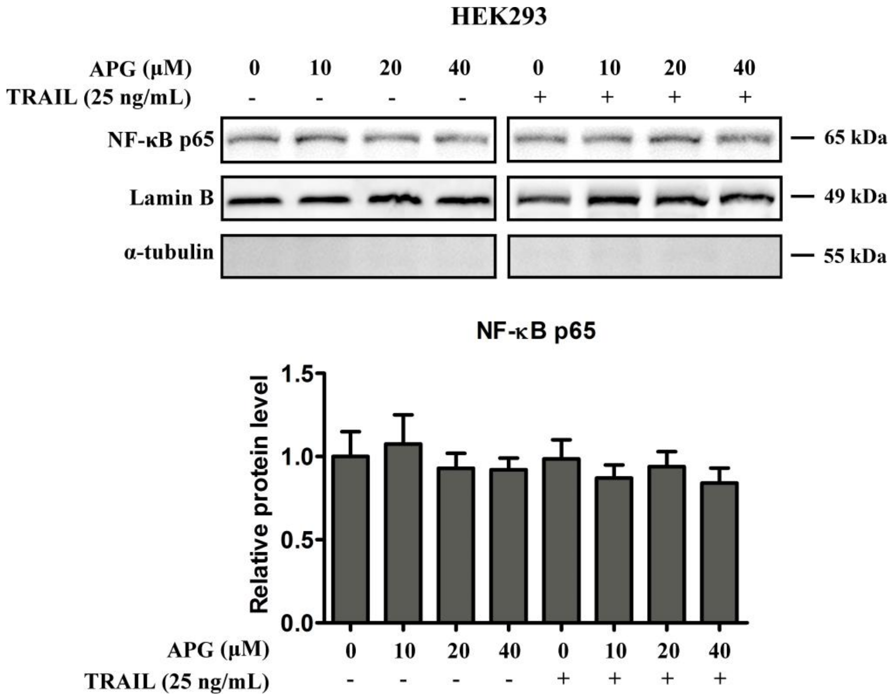

Supplementary Figure S4. Effects of APG and TRAIL on NF-κB level in HEK293 cells. HEK293 cells were treated with APG at different concentrations (0, 10, 20, 40 μM) in the absence or the presence of TRAIL (25 ng/mL) for 24 h. Nuclear proteins were extracted and subjected to Western blotting for p65 detection. Lamin B was used as loading control. All gels run under the same experimental conditions and the representative images of three different experiments were cropped and shown. Densitometric quantification of the immunoblot data is also shown and data are represented as mean ± SD.

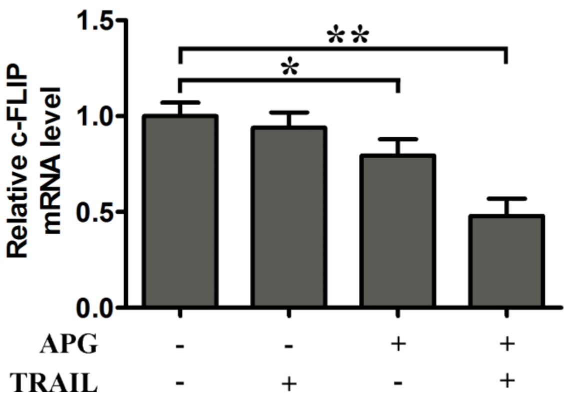

Supplementary Figure S5. Effect of APG and TRAIL on c-FLIP mRNA level. A549 cells were treated with APG (20 μM) in the absence or the presence of TRAIL (25 ng/mL) for 24 h. Q-PCR analysis was performed to detect the level of the mRNA transcripts of c-FLIP. The results shown are representative of three independent experiments. The histogram shows the mean ± SD. *$p < 0.05$, **$p < 0.01$.

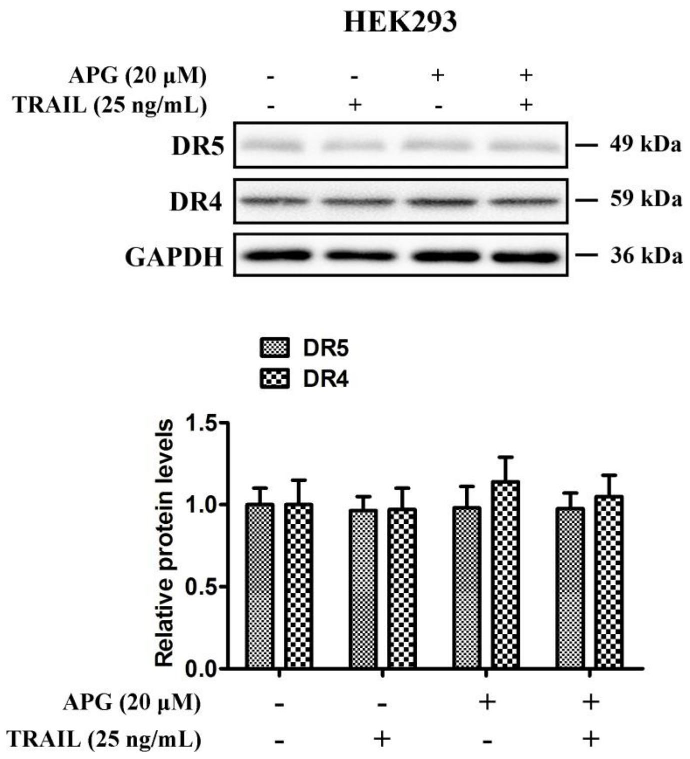

Supplementary Figure S6. Effect of APG and TRAIL on DR4 and DR5 levels in HEK293 cells. HEK293 cells were treated with APG (20 μM) in the absence or the presence of TRAIL (25 ng/mL) for 24 h. Western blotting was performed to detect the levels of DR4 and DR5, respectively. All gels run under the same experimental conditions and the representative images of three different experiments were cropped and shown. Densitometric quantification of the immunoblot data is also shown and data are represented as mean ± SD.

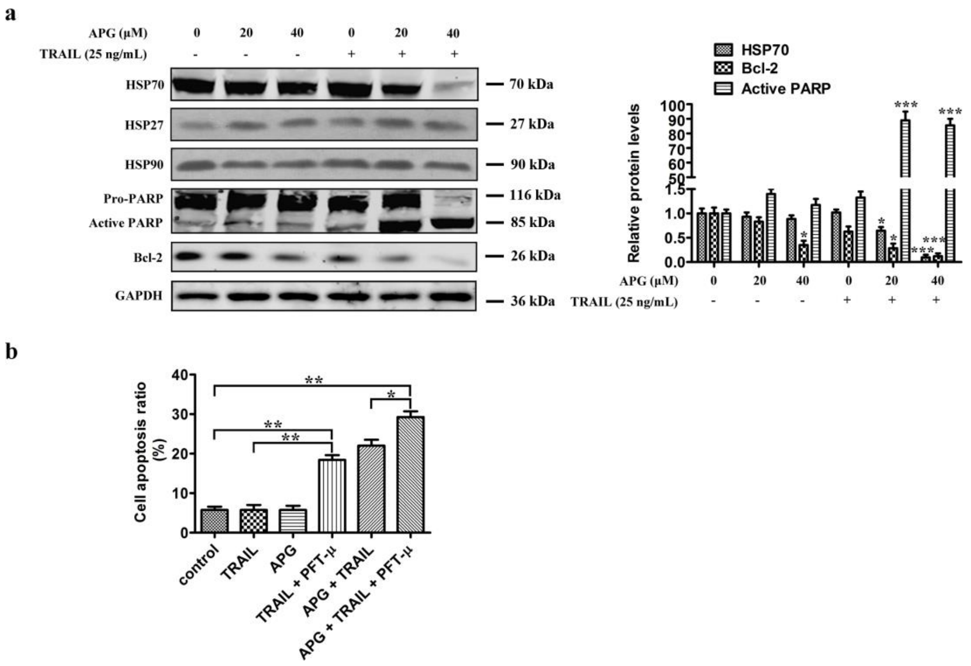

Supplementary Figure S7. Effects of APG and TRAIL on the expression of heat shock proteins. (**a**) A549 cells were treated with APG at different concentrations (0, 20, 40 μM) in the absence or the presence of TRAIL (25 ng/mL) for 24 h. Western blotting was performed to detect the levels of HSP70, HSP27, HSP90, Bcl-2, and PARP, respectively. All gels run under the same experimental conditions and the representative images of three different experiments were cropped and shown. Densitometric quantification of the immunoblot data is also shown and data are represented as mean ± SD. *p < 0.05, ***p < 0.001. (**b**) A549 cells were treated with APG (20 μM), TRAIL (25 ng/mL), PFT-μ (5 μM), or their combination for 24 h before determination of cell death by flow cytometry analysis. Data are representative of three independent experiments and are represented as mean ± SD. *p < 0.05, **p < 0.01.

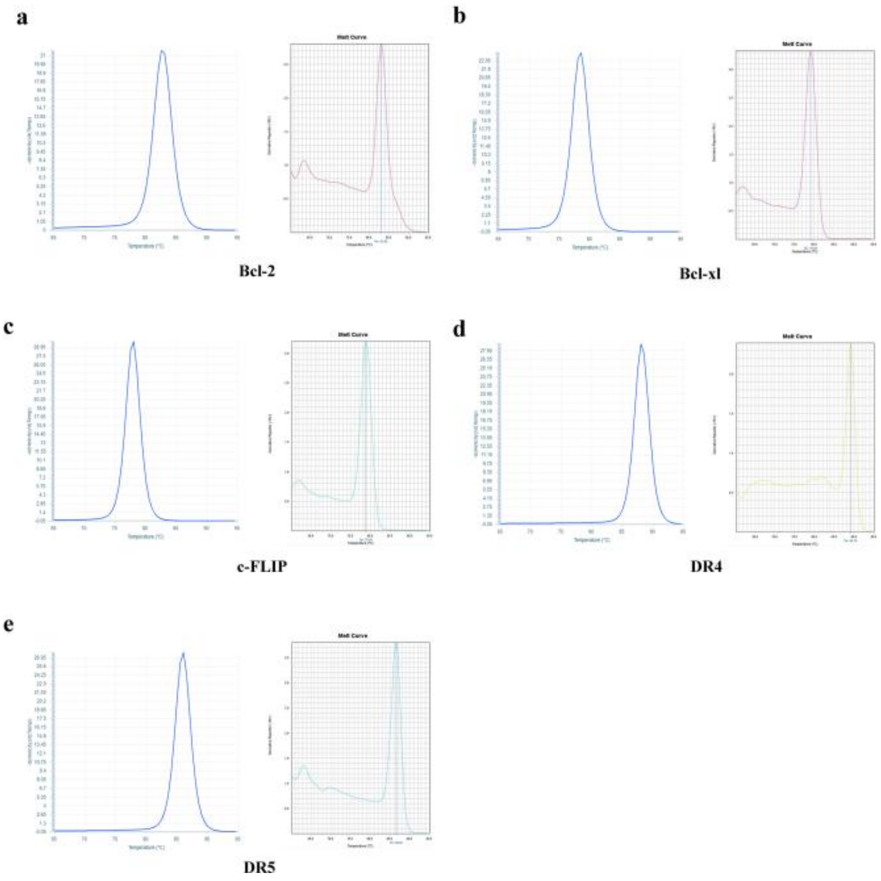

Supplementary Figure S8. The thermal dissociation curve of all the primers pairs, including Bcl-2 (**a**), Bcl-xl (**b**), c-FLIP (**c**), DR4 (**d**) and DR5 (**e**), are shown in comparison with the predicted dissociation curves from uMelt (https://dna.utah.edu/umelt/umelt.html) for the same primers pairs.
